# Proprioceptive Estimation of Forces Using Underactuated Fingers for Robot-Initiated pHRI

**DOI:** 10.3390/s20102863

**Published:** 2020-05-18

**Authors:** Joaquin Ballesteros, Francisco Pastor, Jesús M. Gómez-de-Gabriel, Juan M. Gandarias, Alfonso J. García-Cerezo, Cristina Urdiales

**Affiliations:** 1 Department of Computer Languages and Science, University of Malaga, Escuela de Ingeniería Informática, 29071 Málaga, Spain; 2 Systems Engineering and Automation Department, University of Malaga, Escuela de Ingenierías Industriales, 29071 Málaga, Spain; fpastor@uma.es (F.P.); jesus.gomez@uma.es (J.M.G.-d.-G.); jmgandarias@uma.es (J.M.G.); ajgarcia@uma.es (A.J.G.-C.); 3 Electronics Technology Department, University of Malaga, Escuela de Ingeniería Telecomunicación, 29071 Málaga, Spain; acurdiales@uma.es

**Keywords:** physical Human–Robot Interaction, force estimation, underactuated grippers, adaptation

## Abstract

In physical Human–Robot Interaction (pHRI), forces exerted by humans need to be estimated to accommodate robot commands to human constraints, preferences, and needs. This paper presents a method for the estimation of the interaction forces between a human and a robot using a gripper with proprioceptive sensing. Specifically, we measure forces exerted by a human limb grabbed by an underactuated gripper in a frontal plane using only the gripper’s own sensors. This is achieved via a regression method, trained with experimental data from the values of the phalanx angles and actuator signals. The proposed method is intended for adaptive shared control in limb manipulation. Although adding force sensors provides better performance, the results obtained are accurate enough for this application. This approach requires no additional hardware: it relies uniquely on the gripper motor feedback—current, position and torque—and joint angles. Also, it is computationally cheap, so processing times are low enough to allow continuous human-adapted pHRI for shared control.

## 1. Introduction

Recent trends in robotics pursue the incorporation of robotic systems among people. Social robots are taking on increasing importance, mostly for healthcare applications, i.e., helping patients [1] or elderly people [2]. Collaborative robots (i.e., cobots) are expected to cooperate in physical tasks with them (e.g., moving large objects [3]). Cooperation requires adaptation on both sides. Hence, cobots must be safe [4,5,6] and include force-sensing capabilities to better adapt to people’s feedback and constraints.

Force sensing is particularly important in physical Human–Robot Interaction (pHRI), where robots are expected to physically manipulate a person, e.g., during rehabilitation [7], or when using exoskeletons [8] or prosthesis [9]. In these cases, dependability and safety become a major concern [10,11], especially when it is up to the robot to intentionally touch and/or manipulate people using grippers [12]. These applications include assistive robotics [13], search and rescue missions [14] and healthcare applications [15] among others. These robots need to accommodate to humans’ constraints and needs via shared control. To achieve continuous, transparent adaptation, low level shared control must rely on blending the robot commands with human intention. Although accuracy in force estimation is not critical at low level, as no high precision is required and constant feedback and adaptation tend to correct minor errors, it is at least necessary to assess human force direction and magnitude, so the robot can comply with the person’s input.

There are several methods to estimate human/robot interaction forces. The most common ones are industrial force/torque sensors, current-controlled cobots and sensitive manipulators with joint torque sensors. Industrial sensors are accurate and provide Cartesian external forces, but they are usually bulky, heavy and expensive. Manipulators with joint torque sensing can be implemented using elastic joints, which can be easily developed using the position error and the arm inverse Jacobian [16], but provide poor performance. Motor current-based force estimation depends on an accurate model of the arm dynamics with frictions [17]. Some other robots include rigid joints with torque sensors integrated with the controller, used to provide impedance control. Those robots are expensive and require complex control [18], and they are used mostly for research purposes. Some experimental force sensors for robot arms are based on lightweight pressure sensors such as piezoresistive sensors [19], MEMS barometric sensors [20], or optical sensors [21], which are still under development.

Alternatively, force estimation could be performed on end effectors instead of on the robot arm. The actual choice of the end-effector is important for safe human manipulation. Although there are many different grippers [22], safe, reliable and autonomous grippers, sensitive enough to manipulate human limbs are still under research. Soft grippers are receiving a growing interest in this field [23], but precise manipulation often requires an adaptive but more controllable solution. Gripper-based proprioceptive sensing has already been used in pHRI and can provide a more controllable solution. In [24], variations on the forearm perimeter have been used to estimate hand postures. Some studies [25] propose estimating forces using uniquely proprioceptive sensors in an arm to estimate the position of a surgical instrument carried by an underactuated arm (continuous flexible or made of rigid serial links) based on the actual positions of the intermediate stages, and also the interaction forces.

The authors have already published works in the field of force sensing in pHRI [26] and intelligent tactile perception in robotics [27]. Specifically, in [28] they have suggested using additional joint angle sensors to obtain shape estimation on grasped objects for limb manipulation planning. In this work we propose extending the capacities of the gripper in [28] to also assess forces exerted by the human forearm with no further modifications, i.e., additional sensors. Rather than building an analytic model, given the complexity of these problems, many of these methods rely on machine learning techniques. For example, in [29], interaction forces between a human and a cobot were measured using an external industrial 6-axis force sensor. In [30], the interaction force is predicted by using flexible joints with integrated force sensors, to perform an estimation of the arm global friction, but the approach requires additional functional and fully calibrated force sensors in the gripper.

**Figure 1:**
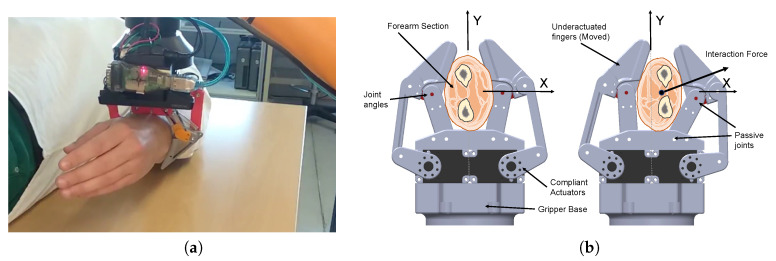
The proposed approach estimates the forces applied by a human in a frontal plane when the forearm is grasped by a robot (**a**) with an underactuated gripper using only the proprioceptive information from servos and passive joint angles (**b**).

In this work, we propose a regression model to detect interaction forces in a gripper with two underactuated fingers with two-phalanx using uniquely its own proprioceptive joint sensors, namely servo and one passive joint angle. Specifically, the gripper is expected to manipulate the forearm of a person in a frontal plane. The gripper adapts to the human’s forearm by design, flexing fingers and shifting forces to keep the grip. As the person moves to either comply with the robot motion or resist, finger angles and applied torque keep adapting, implicitly providing information on the direction and magnitude of human forces on the grip, as illustrated in Figure 1. The ultimate goal of our proposal is to obtain information on human intention and interaction force in a continuous way so that the robot may adapt to human needs and constrains in a transparent way via shared control in applications involving pHRI. The main novelty of our proposal is that it relies on information that grippers can provide without any additional hardware. Hence, we avoid any extra weight, cost and/or complexity in the system. Due to the non-rigid properties of the human forearm and the use of machine learning methods, proprioceptive sensor information (servo and passive articulation positions) is related to forces. Hence, we can train a model using data from tests with volunteers where forces are measured using independent sensors. Thus, inexpensive underactuated grippers with different number of fingers can be used to estimate human intention for efficient, low level shared control in assistive robotics.

The paper is structured as follows. Section 2 presents the design of our gripper and its control system, as well as its kinematic and dynamic analysis. Section 3 describes the tests done and the results obtained. Section 4 presents the experimental setup of the system. Finally, these results are discussed in Section 5, and conclusions and future work are provided in Section 6.

## 2. The Underactuated Gripper

This section presents our gripper design along with a kinematic and dynamic analysis. Moreover, the experimental prototype and the sensing and control systems are described.

### 2.1. Design

**Figure 2:**
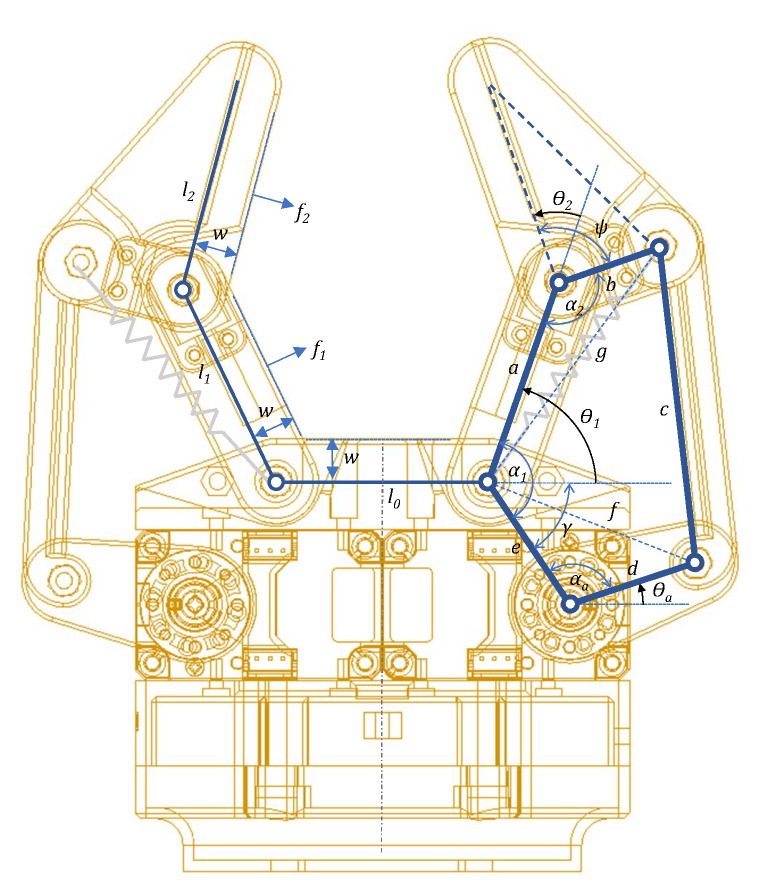
Kinematic design of the gripper for pHRI showing the parameters and joint angles. For clarity, every finger has been partially labeled.

The design proposed in this paper consists of a gripper with two independent underactuated fingers, with two phalanxes and a single actuator each as shown in Figure 2. Actuators can be implemented using tendons or rigid linkages. The use of tendons (e.g., Yale OpenHand) as in [31] has been discarded here due to the displacement of the internal contact surfaces of the fingers when pinching the human skin. Hence, our gripper relies on a transmission system based on rigid linkages, which also provides a more human-friendly contact.

A special feature of this design is the addition of a joint angular sensor that provides information on the values of the passive joints. This allows us to evaluate how the grip adapts to a human’s forearm. A prototype has been manufactured using FDM 3D-printers, and the CAD files have been released openly in a public repository (https://github.com/TaISLab/umahand).

### 2.2. Forward Kinematics

As the value of the joint positions in the adaptive fingers depends on the interaction forces with the environment, they provide information about the shape of the contact surfaces. The value of $\theta_{2}$ ($\theta_{2l}$ and $\theta_{2r}$ for left and right fingers, respectively) is obtained by miniature potentiometers that measure the relative angle between the two phalanxes. The values of $\theta_{a}$ are obtained from the smart servo controllers. Knowing both values, the five-bar mechanism, with fixed-length links, can be solved using trigonometric methods, so the angle of the first phalanx $\theta_{1}$ can be computed.

Solving the trigonometric equations and using the auxiliary angles $\alpha_{1}=\theta_{1}+\gamma$, $\alpha_{2}=\pi -\psi +\theta_{2}$ and $\alpha_{a}=\pi -\theta_{a}-\gamma$, the forward kinematics model is presented in Equation (1).

(1) $$\theta_{1}=\mathrm{asin}\left(\frac{d}{f}\;\sin\left(\theta_{a}+\gamma \right)\right)+\mathrm{asin}\left(\frac{b}{g}\;\sin\left(\psi -\theta_{2}\right)\right)+\mathrm{acos}\left(\frac{f^{2}+g^{2}-c^{2}}{f\;g}\right)-\gamma$$

where *f* and *g* are the non-adjacent vertex distances indicated in Figure 2, computed as (2) and (3).

(2) $$f=\sqrt{d^{2}+e^{2}+d\;e\;\cos\left(\theta_{a}+\gamma \right)}$$

(3) $$g=\sqrt{a^{2}+b^{2}+a\;b\;\cos\left(\psi -\theta_{2}\right)}$$

### 2.3. Dynamic Model

The general dynamic model for each of the rigid linkage-driven underactuated fingers, with multiple DOFs is given by

(4) $$M\left(\theta \right)\;\ddot{\theta }+C\left(\theta ,\dot{\theta }\right)\;\dot{\theta }+G\left(\theta \right)+F\left(\dot{\theta }\right)=T^{T}\left(\theta \right)\;\tau_{a}+J_{1}^{T}\left(\theta \right)\;F_{ext_{1}}+J_{2}^{T}\left(\theta \right)\;F_{ext_{2}}$$

where $\theta ={\left[\theta_{1}\;\theta_{2}\right]}^{T}$ is the 2×1 joint vector for each finger composed by the values of joint 1 and 2. $\ddot{\theta }$, $\dot{\theta }$ denote its acceleration and velocity vectors respectively. M(θ) represents the 2×2 symmetric positive definite inertia matrix, $C(\theta ,\dot{\theta })$ the Coriolis and centripetal torques matrix, and G(θ) and $F(\dot{\theta })$ the 2×1 gravity and friction vectors. $\tau_{a}$ is the scalar actuator torque, $T^{T}\left(\theta \right)$ is the 2×1 transposed transfer matrix that relates the velocities of the actuators to the joint velocities, $J_{1}^{T}\left(\theta \right)$ and $J_{2}^{T}\left(\theta \right)$ are the 2×2 transposed Jacobian matrices of the contact points on phalanx 1 and 2 respectively, where the corresponding Cartesian forces $F_{ext_{1}}$ and $F_{ext_{2}}$ are considered. As all the motion axes are parallel, forces in other directions are rejected by the planar kinematic constraints. This way, Cartesian forces are expressed as a 2×1 vector of two coordinates along the finger plane.

The actuator torque $\tau_{a}$ is provided by a servomotor that has its own dynamics (5) and follows a proportional position control law with torque limitations, as in (6), which renders the finger compliant.

(5) $$\tau_{a}=\tau_{m}-J_{m}\;{\ddot{\theta }}_{a}-B_{m}\;{\dot{\theta }}_{a}$$

(6) $$|\tau_{m}|=min\left\{|\left(\theta_{closed}-\theta_{a}\right)K_{p}|\;,\tau_{max}\right\}$$

where the actual position actuator is $\theta_{a}$, and $J_{m}$ and $B_{m}$ are the moment of inertia and frictions of the servomotor respectively, which have to be taken into account, as its gear box has four spur gears with a 350:1 ratio. The motor torque $\tau_{m}$ is computed at the embedded controller based on the following fixed parameters: reference value for the closing position $\theta_{closed}$, proportional gain $K_{p}$ that defines the compliance, and maximum torque $\tau_{max}$ that limits the grasping force.

## 3. Force Estimation Method

**Figure 3:**
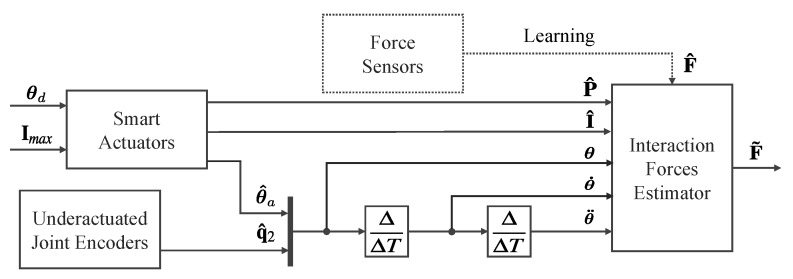
Representative schematic of the intelligent perception system. The regression model uses the measurements from the proprioceptive sensors of the smart actuators and the underactuated joints to estimate external forces. The dotted line represents the supervised learning process, which uses ground-truth forces measured with force sensors for training.

To overcome the difficulty of obtaining an analytic model, we propose using regression methods. The proposed method adapts better to imperfections, sensor and mechanical errors and can be extensible to other similar grippers via training. A schematic of the method is showed in Figure 3, where the symbol $\left(\;\hat{\;}\;\right)$ represents measurements, and $\tilde{F}$ are the estimated external forces.

The inputs of the smart actuators’ current-based controllers are the desired position ($\theta_{d}$) and maximum current ($I_{max}$). Current positions (θ), velocities ($\dot{\theta }$) and accelerations ($\ddot{\theta }$) of the joints are considered inputs of the estimator, along with the current ($\hat{I}$) and the PWM of the control signal ($\hat{P}$). The position is measured from the encoders of the smart actuators (${\hat{\theta }}_{a}$) and the sensors integrated in the passive joints ${\hat{\theta }}_{2}$. Velocities and accelerations are computed from the position with discrete derivatives according to the sample time (ΔT). $\hat{P}$ and $\hat{I}$ variables present low correlation in our actuators (Pearson coefficient equal to 0.4379) due to dynamic behavior of the DC motors. Hence, they are kept as input parameters in our model. All the signals are defined for the two-finger gripper as defined in Equation (7), where sub-index *r* and *l* refer to right and left actuator, respectively.

(7) $$\hat{P}=\left[\begin{array}{c} {\hat{p}}_{r}\\ {\hat{p}}_{l} \end{array}\right];\;\;\hat{I}=\left[\begin{array}{c} {\hat{i}}_{r}\\ {\hat{i}}_{l} \end{array}\right];\;\;\theta =\left[\begin{array}{c} \theta_{ar}\\ \theta_{2r}\\ \theta_{al}\\ \theta_{2l} \end{array}\right];\;\;\dot{\theta }=\frac{\Delta \theta }{\Delta T};\;\;\ddot{\theta }=\frac{\Delta \dot{\theta }}{\Delta T};\;\;\hat{F}=\left[\begin{array}{c} {\hat{f}}_{x}\\ {\hat{f}}_{y} \end{array}\right];\;\;\tilde{F}=\left[\begin{array}{c} {\tilde{f}}_{x}\\ {\tilde{f}}_{y} \end{array}\right]$$

Thus, the goal is to find a non-linear function (L). According to Equation (8), this function estimates external forces from input parameters. We propose two regression methods to obtain L: (i) Support Vector Regression (SVR) [32]; and (ii) Random Forest Regression (RFR) [33].

(8) $$\tilde{F}=L\left(\hat{P},\hat{I},\theta ,\dot{\theta },\ddot{\theta }\right)$$

SVR relies on fitting the error rate within a certain threshold rather than minimizing it (Principle of Maximal Margin). The main advantages of SVR is that it is a non-parametric technique, i.e., it does not depend on distributions of the underlying dependent and independent variables. Additionally, it allows for construction of a non-linear model without changing the explanatory variables, helping in a better interpretation of the resultant model. RFR is a type of additive model that makes predictions by combining decisions from a sequence of base models (ensemble learning), where each based classifier is a decision tree. Unlike linear models, RFR can capture non-linear interaction between the features and the target. Both methods are appropriate to work with non-linearity and outliers, so they are good candidates to solve our problem.

## 4. Experimental Setup

By adding proprioceptive angular sensors, the angles $\theta_{2l}$ and $\theta_{2r}$ can be measured (we use _*l*_ and _*r*_ subscripts for left and right fingers). Thus, given the position information provided by the servos ($\theta_{al}$ and $\theta_{ar}$), the position of the remaining phalanxes ($\theta_{1l}$ and $\theta_{1r}$) can be computed. As a result, the position of the gripped objects can be estimated using $\theta_{2r}$ and the shape of the grasped object can be inferred with the positions of the two fingers $\theta_{l}$ and $\theta_{r}$.

Two potentiometers (*muRata* SV01 10 kΩ linear) have been used for the measurement of the distal joints. They have been added to the gripper as a DAQ with a 50 Hz sample rate. The actuators are *Dynamixel XL450-W250* servos featuring a 12-bit digital magnetic encoder ($0.088^{\circ }$ resolution), and an advanced position-based controller with torque limits to provide a sort of force control. This capability is essential to control the grasping force. Their internal position PID loops have been set to Proportional-only control to get compliance to the user interaction forces. The servos provide real-time feedback of the positions ($\theta_{a}$), the electrical current (*I*), and the PWM output (*P*) of the controller.

A microcontroller board (Arduino Mega 2560) has been used as gripper interface and DAQ that periodically samples the analog values with 10-bit ADC ($0.26^{\circ }$ resolution), from the potentiometers, and queries the status of the servos at a rate of 10Hz. A serial port over USB communicates with the main computer to provide the above information and receives simple Open/Close commands.

The actual values of the parameters for the kinematics shown in Figure 2 can be found in Table 1. It has been designed to grasp an upper forearm with a perimeter between 16.2 and 19.3 cm and a median of 17.7 cm, according to the anthropometrics from [34].

The joint ranges present different mechanical limits. In particular, $0\geq \theta_{2}\geq \pi /2$. The position of the phalanxes depends on the balance between external forces $F_{1}$, $F_{2}$, the actuator torque $\theta_{a}$ and the extension springs (164N/m) used to make the finger stable when no external forces are applied.

**Figure 4:**
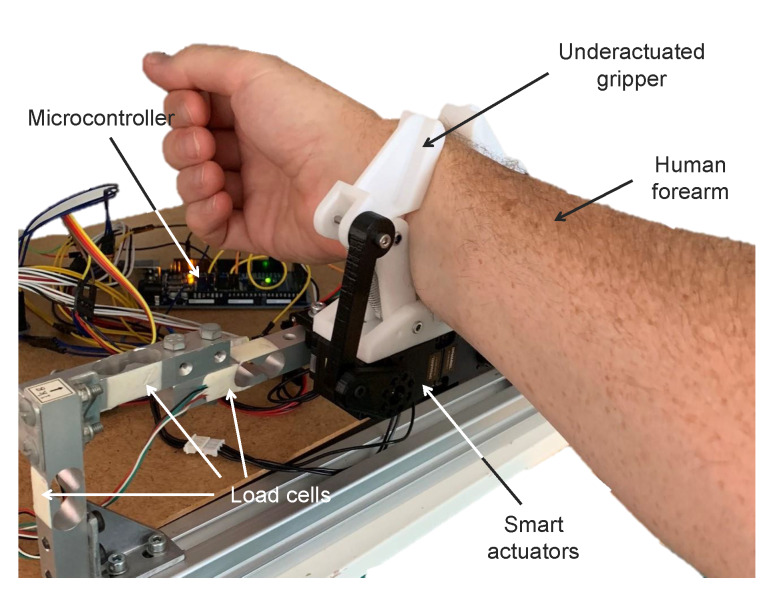
Illustration of the data collection process with the experimental force-sensing system (left side visible only) to record ground-truth data and gripper readings to train the regression methods. Please note that only three load cells (left finger) are visible in this picture as the other three (right finger) are hidden by the human forearm.

To use machine learning techniques to estimate forces using the gripper, we need to capture all the problem instance parameters simultaneously. Also, we need a test environment that ensures repeatability. Hence, we have designed the structure in Figure 4 to obtain a ground-truth for our regression methods. It can be observed that the gripper is physically attached to six load cells used to estimate Cartesian forces. Thus, when the gripper is closed around a moving forearm, we can record its force and all gripper parameters at the same rate of 20 Hz.

**Figure 5:**
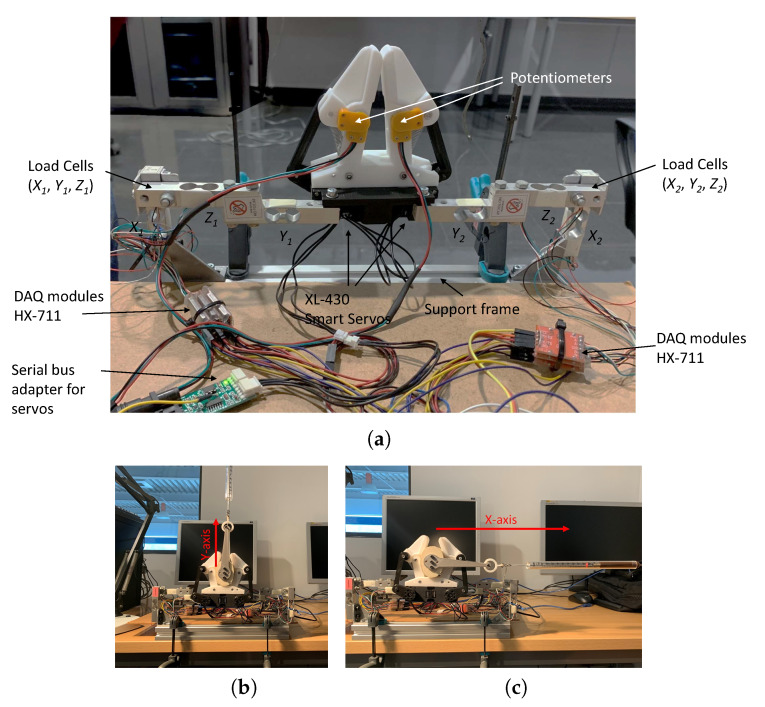
Experimental setup (**a**) and calibration process for Y-axis (**b**) and X-axis (**c**) forces. A dummy forearm section is used to calibrate the force sensor used to get ground-truth values for the force estimation methods.

Force sensors in our structure (see Figure 5a) have been calibrated using a dummy forearm section and a force meter to obtain ground-truth values. Different forces at different angles have been applied (Figure 5b) and recorded together with its corresponding load-cell readings. Then, a matrix that relates the six readings from the load cells has been adjusted using a least squares method. The inverse of this matrix will then be used to provide output forces in kgf units from load-cell readings.

After the calibration of the force sensor, the positions of the fingers were controlled—proportional control—to maintain the person’s forearm as close as possible to the center of the gripper. We purposefully kept a low gain in our control loop to make it more sensitive to external perturbations.

## 5. Experimental Protocol and Results

To test our system, five volunteers were asked to get their forearms gripped and pull in a frontal plane (X and Y movements) to obtain a training data set. Each volunteer performs 2-min circular motion and 2-min cross motion Forces and gripper parameters were captured in a continuous way with a common time reference at 20 Hz (generating more than 20,000 samples).

**Figure 6:**
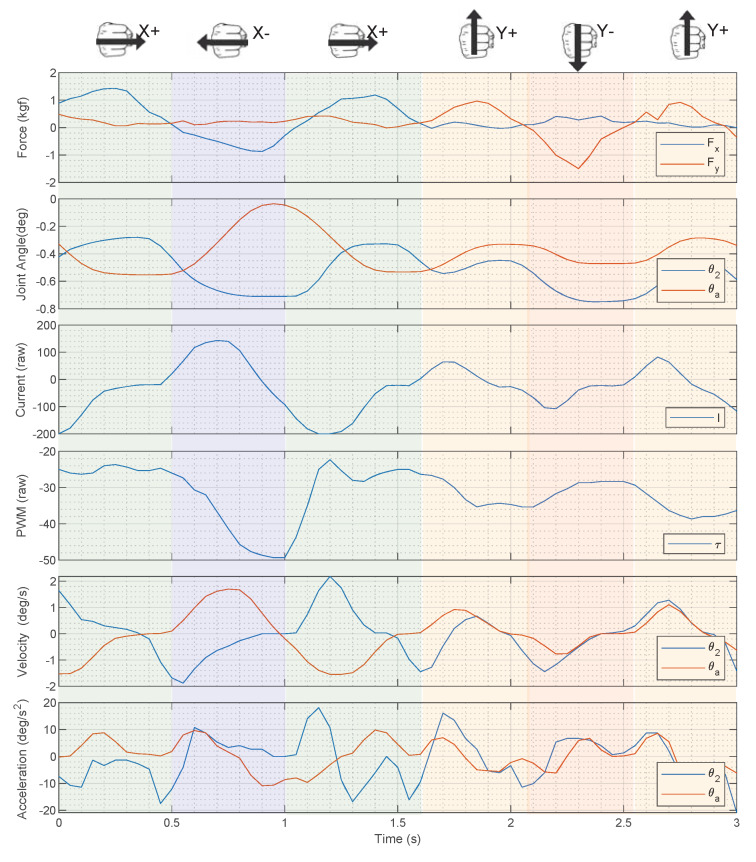
Excerpt from the data collected during experiments: exerted forces ($F_{x}$, $F_{y}$) and the input parameters position ($\theta_{2}$, $\theta_{a}$), current (*I*), PWM (*P*), velocity (${\dot{\theta }}_{2}$, ${\dot{\theta }}_{a}$), and acceleration (${\ddot{\theta }}_{2}$, ${\ddot{\theta }}_{a}$), for left finger. Right finger data are analogous.

Figure 6 shows forces measured in the X and Y axes by the load cells in the structure, the corresponding joint and servo positions and the current and PWM provided by the servo controller for the left gripper side. Signals may be positive or negative depending on the motor direction. In this example, the person first moves his forearm 3 times right and 3 times left (X-axis) and then 2 times up and 2 times down (Y-axis) in a sequence. Forces during the sequence can be clearly observed as peaks in the X axes in Figure 6. It can also be observed how $\theta_{a}$ and $\theta_{2}$ evolve with the forces. Similarly, the current and PWM also present changes depending on forces. It can be observed that in general, forces in the x-axis (e.g., seconds 1 to 3.5) are more correlated with the gripper parameters than forces in the y-axis (e.g., seconds 3.5 to 5). This was expected because the gripper is aligned with the Y-axis in our tests, so the gripper fingers tend to slip when forearms move in vertical.

As commented in Section 2.3, dynamic parameters are required for force estimation. We can obtain angular velocities and acceleration extracting the first and second derivatives of $\theta_{a}$, $\theta_{2}$. To reduce noise in these new features, we use a moving average filter (size 3). Figure 6 shows these first and second derivatives, which present similar trends with exerted forces.

It can be observed that the derivatives obtained are centered in zero, whereas $\theta_{a}$, $\theta_{2}$ are not. This happens because these angles depend on the gripped forearm shape. To avoid this shape dependence, we propose using the initial forearm angles $\theta_{a}$, $\theta_{2}$, obtained when the gripped initially closes, as an offset.

### 5.1. Data Modelling

Our problem requires multi-variable analysis as we need to estimate forces on the X and Y-axis. The first decision before modelling was consequently whether to work with forces independently or together. We have decided to model them independently; otherwise, any motion patterns in training sets presenting casual relationships in X and Y, e.g., users doing circular motion or favoring one side against the other, would be acquired in the model.

As commented, we have tested RFR and SVR to create our model. Both techniques are appropriate to deal with non-linearity (e.g., Figure 6). RFR is particularly fit to cope with problems where parameters have different importance depending on the situation. For example, when forces in the x-axis are low, the current parameter (*I*) does not provide much information, whereas $\theta_{2}$ still correlates with the force (Figure 6). However, SVR deals better than RFR with sparse data, which are present in boundaries—i.e., large forces—in our data set.

MATLAB (Version used: R2019b by The MathWorks, Natick, MA, USA) has been used to create RFR and SVR models. RFR has been created using the TreeBagger function. The best hyperparameter set for our data was: NumTrees = 40, MinLeafSize = 5. SVR was created with the fitrsvm function. Kernel radial with standard parameters outperforms linear and polynomial kernel for our data. Automatic parameter optimization (OptimizeHyperparameters) has been used to select the best values for BoxConstraint, KernelScale and Epsilon parameters.

### 5.2. Performance Evaluation and Discussion

**Figure 7:**
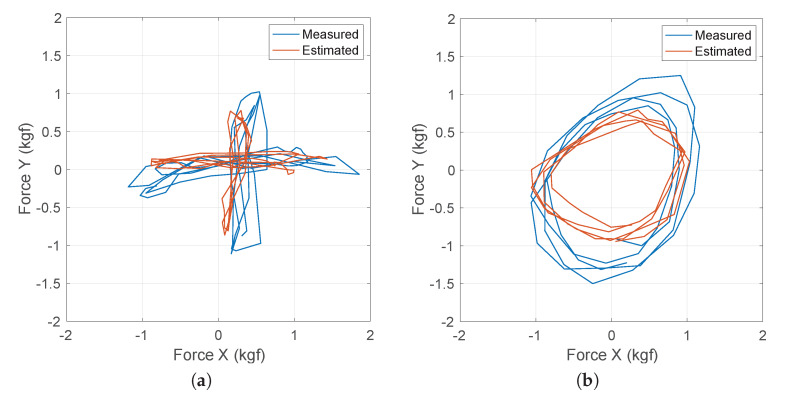
Estimated vs. measured forces for two types of interaction experiments: (**a**) vertical and horizontal forces during 4 s, and (**b**) circular forces trying to describe a circle for 2.8 s.

In this section, we analyze force estimation results in the frontal plane (X and Y), both for RFR and SVR. To do so, we acquired information from 5 volunteers using the described system. Each volunteer performed two tests: (i) exerting forces in a cross pattern (only X or Y direction at a given time), and (ii) exerting forces in a circular pattern (forces both in X and Y at all times), as shown in Figure 7. Volunteers were trained to use constrained forces, although no mechanism was applied to keep them in a constrained interval. Ten tests were recorded per volunteer in a total testing time of 2 min after training. Afterwards, a k-fold cross-validation technique per volunteer (k=5) was used to evaluate the accuracy of the models. Finally, the Mean Absolute Error (MAE) was calculated for all tests of each regression model in X and Y.

**Figure 8:**
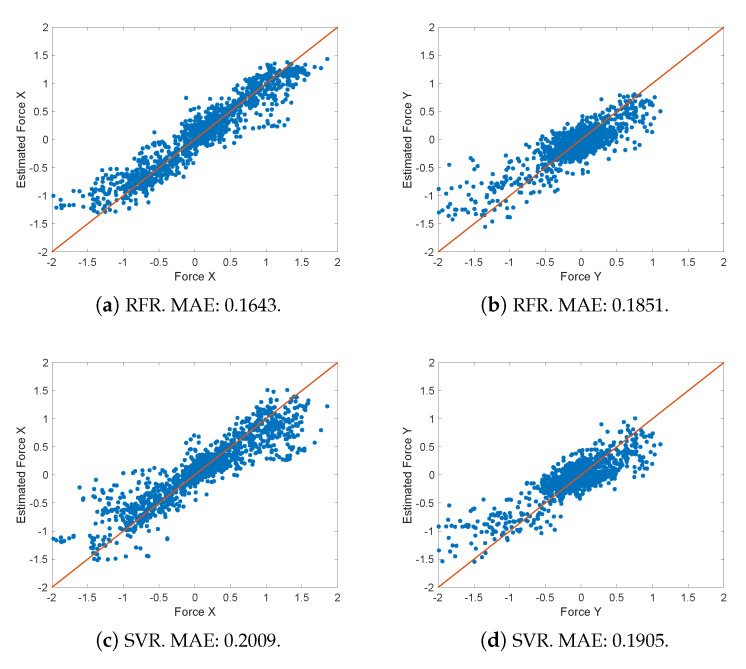
X (**left**) and Y (**right**) real Cartesian forces versus estimated forces using RFR (**top**) and SVR (**bottom**) methods.

Figure 8 shows the resulting Force X and Force Y estimates versus the measured force values for all tests using both methods (SVR and RFR) in terms of MAE. RFR outperforms SVR slightly for our data set. It can be observed that in the middle range both models behave similarly but in all cases RFR provides a better fit for low and high force values. This confirms the importance of input parameters depends on the force ranges. To understand this dependence, we can observe the importance of those input parameters using the OOBPermutedPredictionDeltaError variable from the TreeBagger RFR function in MATLAB (Table 2). It can be observed that current *I* and the servo speed ${\dot{\theta }}_{a}$ are the most relevant parameters to estimate forces in the X-axis. However, to estimate forces in the Y-Axis the second passive joint angle $\theta_{2}$ and its variation become more relevant. This is most likely due to slippage in fingers when users pull their forearm up and/or down, meaning that variations in the grip become more important than forces in the servoes in these cases.

Figure 7 shows estimates versus real force values for a single test over time. It can be observed that the force module tends to be underestimated. This effect is higher in the Y-axis compared to the X-axis (see Figure 7b), due to slippage in the grip. It can be observed that the motion pattern is correctly estimated. Hence, the user’s intention can be estimated from these forces estimation to be used in a shared-control approach, i.e., to adapt emergent motion patterns to the user’s preferred direction. Typically, users’ intention in shared control in pHRI has been obtained in different ways. In [35] intent is defined in a binary way (e.g., motion or not, left or right, up or down, etc.). Also, it is defined within a set of discrete intents (e.g., predefined poses) or using continuous variables (e.g., steering angles). Our approach provides Cartesian X and Y forces. These forces can be transformed into polar coordinates—error module ($\rho_{\epsilon }$) and angle ($\theta_{\epsilon }$)—to calculate the classification accuracy and estimation error in all three typical user intent estimations: binary intent (left or right, i.e., 180 degrees clustering), discrete intents (left, right, up and down, i.e., 90 degrees clustering) and continuous intent (module and angle). Table 3 shows the results. It can be observed that the classification accuracy remains high in binary and discrete cases (above 90%), with values similar to the ones presented in [36]. On the other hand, continuous values provide a granularity below 20 degrees on average in direction, so applications that need to know the user’s intention to collaborate with them in tasks with some degree of freedom—e.g., repositioning a limb vs. (precise) assistive surgery—can rely on the proposed approach.

## 6. Conclusions

In this work, we have presented a method to estimate interaction forces with an underactuated gripper grasping a human forearm. These forces are related to human intention and, hence, critical for pHRI. The intention is typically used in shared-control approaches to ensure that human constraints, goals, and comfort are taken into account while their forearms are being manipulated.

We use only the gripper proprioceptive sensors to estimate the forces. Specifically, we work with a gripper with two underactuated fingers to achieve an adaptive, robust, and precise grasping of human limbs operating in a closed control loop. Its proprioceptive sensors provide information about the servo and passive joint positions—using uniquely two inexpensive potentiometers—, plus the motor PWM and current. The analytical model of the gripper may already produce limited information about human forces in the gripper, but the model is only valid for certain conditions and it does not take into account limb slippage or artifacts. Instead, we propose using machine learning to estimate human forces.

We have designed a platform to capture the required learning data set that includes a fixed gripper and a force-measurement structure to get training data. When volunteers are moving their gripped forearms, we gather all the gripper parameter values as well as the load-cell readings. We tested SVR and RFR to predict forces using the acquired data set. RFR provides slightly better results because it adapts better to the nature of our data: depending on the force range, some input parameters provide more information than others. Specifically, we observed that force in X is better estimated using *I* and ${\dot{\theta }}_{a}$ and force in Y is better estimated using $\theta_{2}$ and ${\dot{\theta }}_{2}$. After training, the method is computationally cheap and resulting trees can be run in parallel.

The proposed method does not require any additional sensor except the gripper proprioceptive ones. Additionally, the proposed proprioceptive sensors are cheap, robust, and do not require calibration for each different gripped object. The gripper has two fingers in the same plane, so only two-dimensional forces (in a frontal plane) are considered because the forces in other directions are rejected by the kinematic constraints of the fingers.

Results prove that the proposed methodology provides satisfactory results in all our tests with different people and changing forces. Future work will focus on developing a gripper with a higher number of non-parallel fingers to consider forces in the full Cartesian space. Also, we will work on implementing shared control based on estimated forces to prove that task efficiency and human comfort improve using the proposed method to estimate user’s intention.

## Figures and Tables

**Table 1:** Values of the parameters of the kinematic model of the underactuated gripper described in Figure 2.

Parameter	Value	Parameter	Value
*a*	40 mm	*e*	27.8 mm
*b*	20 mm	ψ	90°
*c*	60 mm	γ	56°
*d*	25 mm	*w*	10 mm

**Table 2:** Parameter importance in RFR. The parameters with higher importance are in bold.

	$\theta_{2}$	$\theta_{a}$	*I*	*P*	${\dot{\theta }}_{2}$	${\dot{\theta }}_{a}$	${\ddot{\theta }}_{2}$	${\ddot{\theta }}_{a}$
Force X	1.0921	0.9185	**1.3562**	0.9050	0.8265	**1.3502**	0.7936	0.8949
Force Y	**1.6341**	0.7029	0.7296	0.9385	**1.5958**	0.8571	0.7358	0.7156

**Table 3:** User intent estimation depending on the desired shared-control output.

Intent	Classification Accuracy/Estimation Error			
Binary	99.08% (Right)		98.78% (Left)	
Discrete	96.34% (Right)	97.69% (Up)	94.03% (Left)	95.87% (Down)
Continuous	19.2 degrees ($\theta_{\epsilon }$)		0.22 kgf ($\rho_{\epsilon }$)	
